# Assisted reproductive technology and the risk of preeclampsia: an updated systematic review and meta-analysis

**DOI:** 10.1186/s12884-019-2291-x

**Published:** 2019-05-02

**Authors:** Amir Almasi-Hashiani, Reza Omani-Samani, Maryam Mohammadi, Payam Amini, Behnaz Navid, Ahad Alizadeh, Esmaeil Khedmati Morasae, Saman Maroufizadeh

**Affiliations:** 1 Department of Epidemiology, School of Health, Arak University of Medical Sciences, Arak, Iran; 2 Department of Epidemiology and Reproductive Health, Reproductive Epidemiology Research Center, Royan Institute for Reproductive Biomedicine, ACECR, Tehran, Iran; 3 Department of Biostatistics and Epidemiology, School of Public Health, Ahvaz Jundishapur University of Medical Sciences, Ahvaz, Iran; 4 Institute of Psychology, Health, and Society, Department of Health Services Research, University of Liverpool, Liverpool, UK; 5 School of Nursing and Midwifery, Guilan University of Medical Sciences, Rasht, Iran

**Keywords:** Assisted reproductive technology, Preeclampsia, Infertility, Meta-analysis, Systematic review

## Abstract

**Background:**

The objective of this systematic review and meta-analyses was to assess the risk of preeclampsia among women who conceived with assisted reproductive technology (ART).

**Methods:**

We searched the ISI Web of Knowledge, Medline/PubMed, Scopus, and Embase (from inception to May 2017) for English language articles using a list of key words. In addition, reference lists from identified studies and relevant review articles were also searched. Data extraction was performed by two authors, and the study quality was assessed using the Newcastle–Ottawa Scale. Random-effects model meta-analysis was applied to pool the relative risks (RR) across studies.

**Results:**

A total of 48 studies (5 case-control studies and 43 cohort studies) were included in this meta-analysis. The Cochran Q test and I^2^ statistics revealed substantial heterogeneity (Q = 26,313.92, d.f. = 47, *p* < 0.001 and I^2^ = 99.8%). Meta-analysis showed a significant increase in preeclampsia in women who conceived by ART compared with those who conceived spontaneously (RR = 1.71, 95% CI = 1.11–2.62, *p* = 0.015).

**Conclusions:**

The findings of this systematic review indicate that the use of ART treatment is associated with a 1.71-fold increase in preeclampsia.

## Background

Assisted reproductive technologies (ART) are used to treat infertility problems and contain methods in which oocyte and sperm are manipulated in vitro [1]. The use of ART has increased exponentially worldwide and is responsible for over than one million births annually [2, 3]. Having been treated by ART, the women who conceived had numerous adverse outcomes, both for themselves and the infants [3]. Previous studies have demonstrated that ART is associated with small for gestational age infants, preterm delivery, perinatal mortality, preeclampsia (PE), gestational diabetes, placenta previa, placental abruption, and cesarean delivery [4]. Of several adverse pregnancy consequences, hypertensive disorders affect 6–8% of all pregnancies through gestational hypertension and PE [5, 6]. In contrast to spontaneous pregnancy, pregnancies with ART are at an increased risk of PE [7]. It remained unclear whether either ART itself [in vitro fertilization (IVF), intracytoplasmic sperm injection (ICSI), intrauterine insemination (IUI), oocyte donation (OD), or embryo donation (ED)] or maternal risk factors associated with ART (that is, advanced maternal age, obesity, change of partner, longer interval between births, reduced smoking, and chronic hypertension) were related to increased risk of PE [7, 8]. Some studies have shown the probability of the taking of some medications during pregnancy, such as low-dose aspirin, [9] prevents for PE in high-risk women [10–12]. Thus, identifying high-risk women during the early period of gestation will be worthwhile for the prevention and management of the pregnancy complications [13]. Finally, the lack of diagnostic criteria for pregnancy complications associated with hypertension, especially for PE, make the research in this field more complicated [14].

In the present paper, the authors conducted a comprehensive systematic review of ART procedures and PE. The aim of this review was to investigate whether ART mediated pregnancies (i.e., IUI, IVF, ICSI, OD, and ED) increased the incidence of PE in pregnancy compared with spontaneous pregnancies.

## Methods

### Search strategy

This meta-analysis was performed according to the PRISMA (Preferred Reporting Items for Systematic Reviews and Meta-Analyses) checklist [15]. We conducted a systematic literature search in Medline/PubMed, Embase, Scopus, and the ISI Web of Knowledge from inception through June 2017 for studies examining the association between ART and PE. In addition, reference lists from all retrieved papers were checked. Table 1 provides more details about the search strategy.

**Table 1:** Search strategy for MEDLINE (MeSH, Medical Subject Headings)

1	Preeclampsia [Text Word])
2	Pre-Eclampsia [Text Word])
3	“Pre-Eclampsia” [Text Word])
4	“Pre-Eclampsia” [MeSH Terms]
5	1 OR 2 OR 3 OR 4
6	Reproductive Techniques, Assisted [Text Word]
7	Reproductive Techniques, Assisted [MeSH Terms]
8	6 OR 7
9	Cohort Studies [Text Word]
10	Cohort Studies [MeSH Terms]
11	Retrospective Studies [Text Word]
12	Retrospective Studies [MeSH Terms]
13	Prospective Studies [Text Word]
14	Prospective Studies [MeSH Terms]
15	Case-Control Studies [Text Word]
16	Case-Control Studies [MeSH Terms]
17	9 OR 10 OR 11 OR 12 OR 13 OR 14 OR 15 OR 16
18	5 AND 8 AND 17

### Inclusion and exclusion criteria

We included published case-control studies and cohort studies evaluating the association between ART and PE risk. No geographic restrictions were used. The following types of studies were excluded: (a) non-English full-text studies, (b) animal studies, (c) repeated or overlapping studies, (d) reviews, meta-analysis and cross-sectional articles, case reports, editorials, and letters to the editor, (e) abstract-only publications or unpublished studies. There were five case-control studies added to the study. However, it was not substantially possible to estimate the relative risk (RR) with case-control design due to the fact that the marginal probabilities were not available; under the rare disease assumption, the odds ratio will be approximate the RR.

### Outcome and exposure

In the present study, all types of ART treatments were considered as the interested exposure variable. Our outcome was PE defined as “elevated blood pressure (BP) (more than 140/100 mmHg) and proteinuria (0.3 g over 24 hours or more).”

### Data extraction

Two authors (MM and SM) independently extracted the following data from all studies meeting the inclusion criteria: first author’s name, year of publication, location, study period, design, sample size, and study results. In addition, outcome data were extracted from each study in a 2 × 2 table, and the results were expressed as RR with their respective 95% confidence intervals (CIs) [9].

### Quality assessment

Two authors (MM and SM) independently assessed the quality of studies using the Newcastle–Ottawa Scale (NOS) [16]. This scale assesses methodology in three domains: (a) selection of study groups, (b) comparability of groups, and (c) ascertainment of exposure and outcomes. Total score ranged from 0 to 9 with a score of ≥8 indicating high quality.

### Statistical analysis

Statistical analysis was conducted using Stata version 13.0 (Stata Corp, College Station, TX, USA). The RR was used as the effect size of association across studies. The Cochran Q test and the I^2^ statistic were used to evaluate heterogeneity among studies [17]. Concerning the Cochrane Q test, *P* < 0.10 was deemed statistically significant for heterogeneity. The I^2^ statistic indicates the percentage of total variation across studies that is due to heterogeneity rather than chance and is classified as mild (25%), moderate (50%), or high (75%) [17]. The Galbraith plot was used to detect the potential sources of heterogeneity [18]. The pooled RR estimate and corresponding 95% CI were calculated by using the random-effect model incorporating between-study variability. The Begg’s rank correlation test, Egger’s weighted regression test, and visual inspection of a funnel plot were used to assess publication bias [19, 20]. All tests were two-tailed and a *P* value of < 0.05 was deemed statistically significant.

## Results

### Study selection

**Fig. 1:**
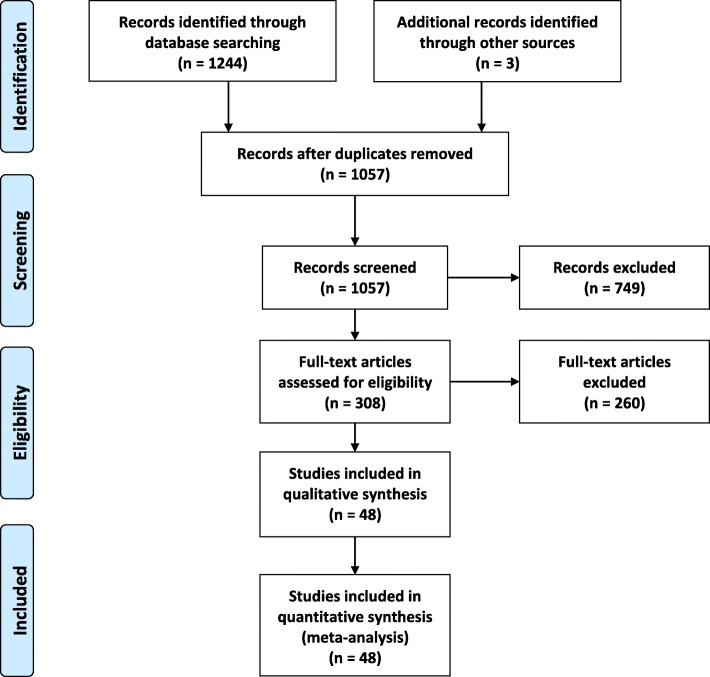
Flow diagram of study process

The process of study selection is illustrated in Fig. 1. A total of 1244 relevant papers were identified using diverse search strategies in four databases (113 from PubMed, 140 from Embase, 897 from Scopus, and 94 from Web of Knowledge) and three records of gray literature. After removing duplicates, 1057 papers remained, and 749 papers were deemed ineligible after title and abstract screening, and 308 relevant papers were considered for further screening through full-text reading. After the exclusion of all non-eligible studies (*n* = 260), a total of 48 studies (5 case-control studies and 43 cohort studies) were included in this meta-analysis.

### Study characteristics

For each study, sample size, total number of ART and non-ART group, number of PE cases in each group, publication date, first author, target country, type of study, and participant mean age of each group were extracted. Cross-sectional studies and non-English studies were excluded from the meta-analysis. All of the primary studies were published between 1999 and 2017 and out of 48 studies, 11 were carried out in the United States, 11 in Asia, and 26 in Europe. The characteristics of studies considered in the meta-analysis are presented in Table 2.

**Table 2:** Characteristics of the primary studies included in the meta-analysis

Author	DOP	Country	Period	Design	PE in ART	ART group	PE in NART	NART group
Julie Hoy [42]	1999	Australia	1982–1995	Cohort	131	1552	399	7717
O.Salha [43]	1999	UK	1992–1997	Cohort	13	112	1	112
A.Geipel [44]	2001	Germany	1995–1999	Cohort	6	114	11	114
Anne Lynch [45]	2002	USA	1994–2000	Cohort	27	198	40	330
Syeda Zaib-un-Nisa [46]	2003	Emirates	1997–2001	Cohort	4	36	4	96
Pinborg [47]	2004	Denmark	1997	Cohort	71	870	49	566
Barbara Luke [48]	2004	USA	1990–2002	Cohort	25	228	24	725
Bengt Kallen [49]	2005	Sweden	1982–2001	Cohort	978	13,261	55,728	2,013,633
Fiona Thomson [50]	2005	Scotland	1989–1999	Cohort	70	1437	556	21,688
Sonia Hernandez-Diaz [51]	2006	USA & Canada	1998–2006	Cohort	18	349	115	4762
Erez [52]	2006	Israel	1988–2002	Cohort	51	292	193	2336
Prefumo [53]	2007	UK	NA	Case Control	1	31	1	62
Apantaku [24]	2008	UK	1999–2004	Cohort	6	88	7	88
Chen [54]	2009	Canada	2005	Cohort	34	1357	77	5190
Sun [55]	2009	Canada	2004–2007	Cohort	31	2118	112	8420
Morcel [56]	2010	France	2001–2005	Cohort	12	104	13	173
Miyake [57]	2010	Japan	2005–2007	Cohort	15	20	111	230
Suzuki [58]	2010	Japan	2000–2007	Cohort	4	64	9	87
Lehnen [28]	2011	Germany	2000–2009	Cohort	10	74	8	305
Yang [59]	2011	Korea	1995–2008	Cohort	9	67	22	143
Kuivasaari-Pirinen [60]	2012	Finland	1996–2007	Cohort	16	255	967	26,870
Bamberg [61]	2012	Germany	1998–2008	Cohort	14	426	24	813
Lubovnik [62]	2012	Slovenia	1997–2009	Case Control	55	246	126	477
Sazonova [63]	2012	Sweden	2002–2006	Cohort	520	11,292	15,984	571,914
Mohammed [64]	2012	Qatar	2002–2011	Cohort	27	145	30	175
Le Ray [65]	2012	France	2008–2010	Cohort	24	144	9	236
Emily Werder [66]	2013	USA	2002–2008	Cohort	45	215	62	232
Sara S. Malchau [67]	2013	Denmark	1995–2010	Cohort	1185	24,305	2519	56,022
Rocio Revello [68]	2013	Italy	2000–1010	Cohort	28	88	14	59
Sari Raisanen [69]	2013	Finland	2006–2010	Cohort	90	5647	3138	285,357
Alex Fong [70]	2014	USA	2009	Case Control	29	551	7487	406,334
Nathan S. Fox [71]	2014	USA	2005–2012	Case Control	61	376	15	137
Tandberg [39]	2014	Norway	1967–2009	Cohort	5516	8549	24,971	493,217
Tali Silberstein [72]	2014	Israel	NA	Cohort	113	1294	7889	171,513
Cagrı Arıoglu Aydın [23]	2015	Istanbul	2007–2010	Cohort	13	137	46	133
Anne-Maude Morency [73]	2015	Canada	2000–2013	Cohort	39	181	4	49
Robert Johnston [27]	2015	USA	2009	Cohort	29	551	7847	406,334
Malinda S. Lee [74]	2015	USA	2006–2008	Cohort	17	108	176	2284
Bay [75]	2016	Denmark	1999–2013	Cohort	2675	30,418	37,531	896,448
DoPierala [76]	2016	UK	1992–2009	Cohort	203	3188	2341	52,443
Nejdet [77]	2016	Sweden	2003–2012	Cohort	1156	27,084	27,912	999,804
Zhu [78]	2016	China	2006–2014	Cohort	98	2641	110	5282
Vikstrom [79]	2016	Sweden	1988–2012	Case Control	607	10,412	822	18,624
Ben-Yaakov [80]	2016	Israel	1988–2012	Cohort	378	4153	4471	95,138
Sun [81]	2016	China	2010–2014	Cohort	42	411	54	742
Valenzuela-Alcaraz [26]	2016	Spain	2004–2010	Cohort	6	488	0	200
Rizzo [82]	2016	Italy	2007–2014	Cohort	17	249	6	260
Guilbaud [25]	2017	France	2010–2014	Cohort	41	303	32	369

*DOP* Date of publication, *PE* Preeclampsia, *ART* Assisted Reproductive Technology, *NART* Non-Assisted Reproductive Technology

### Quantitative data synthesis

**Fig. 2:**
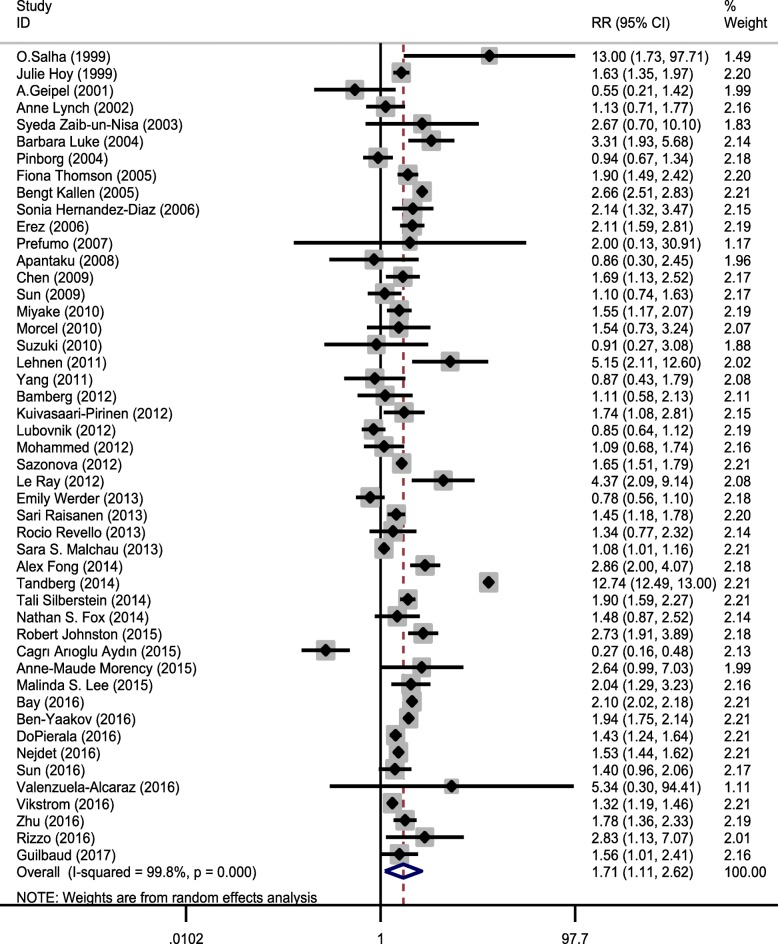
Forest plot showing effect of ART on preeclampsia

A total of 156,246 ART cases (with 14,560 cases of PE) and 6,558,249 non-ART cases (with 202,064 cases of PE) were included in the analysis. Risk ratios and their 95% CIs were reported using the Mantel–Haenszel method. The relationship of ART and the risk of PE were estimated using the 48 primary included studies. The pooled estimate of RR in this meta-analysis revealed that ART was significantly associated with a higher risk of PE (pooled RR = 1.708, 95% CI = 1.111–2.624, z = 2.44, *p* = 0.015), that is, the PE risk in ART group was 1.687 times greater compared to the non-ART group (Fig. 2, Table 3).

**Table 3:** Summary of meta-analysis results and subgroups analysis

Groups	Studies	Test of association				Heterogeneity		
		RR (95% CI)	*P* value	Model	Z	Chi square	*P* value	I square
Total studies	48	1.71 (1.11–2.62)	0.015	Random	2.44	26,313.92	< 0.001	99.8%
Subgroup analyses								
Study design								
Cohort	43	1.73 (1.10–2.72)	0.018	Random	2.36	25,159.19	< 0.001	99.8%
Case control	5	1.46 (0.97–2.20)	0.070	Random	1.81	28.38	< 0.001	85.9%
Time Period								
1999–2010	18	1.64 (1.31–2.05)	< 0.001	Random	4.29	117.09	< 0.001	85.5%
2010–2017	30	1.74 (0.97–3.09)	0.062	Random	1.87	25,671.51	< 0.001	99.9%
Region								
Asia	11	1.71 (1.53–1.92)	< 0.001	Random	9.38	17.12	0.072	41.6%
Europe	26	1.74 (0.95–3.21)	0.075	Random	1.78	25,090.51	< 0.001	99.9%
America	11	1.78 (1.31–2.41)	< 0.001	Random	3.70	52.30	< 0.001	80.9%

*RR* Relative Risk, *CI* Confidence Interval

### Heterogeneity analysis

**Fig. 3:**
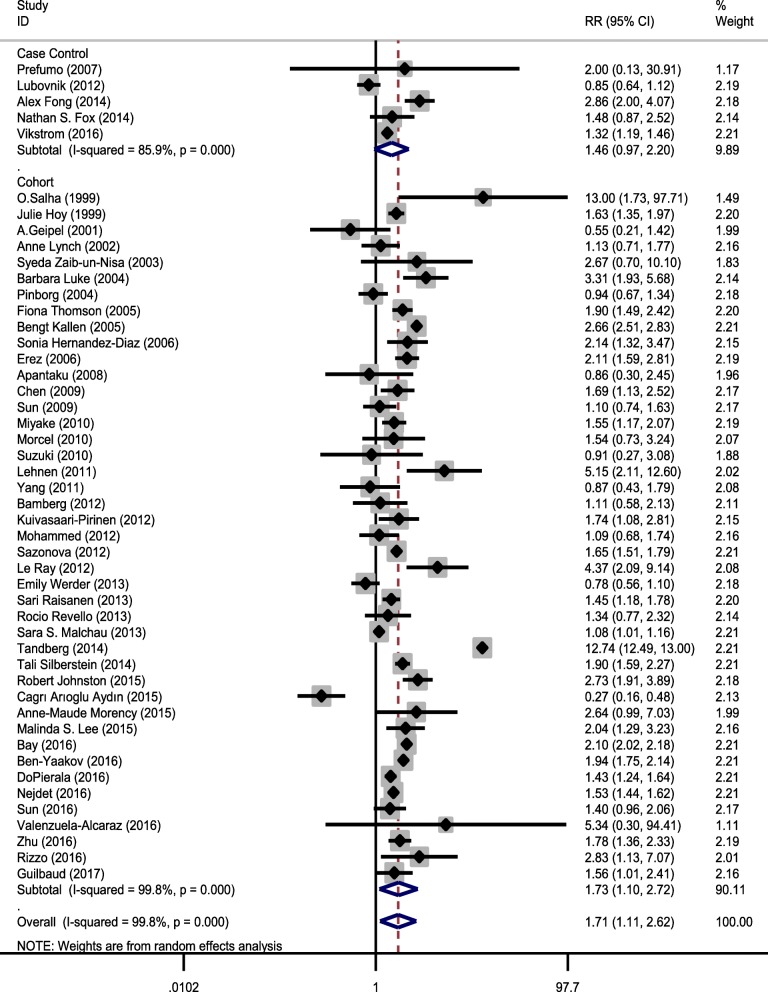
Forest plot showing effect of ART on preeclampsia based on study design

**Fig. 4:**
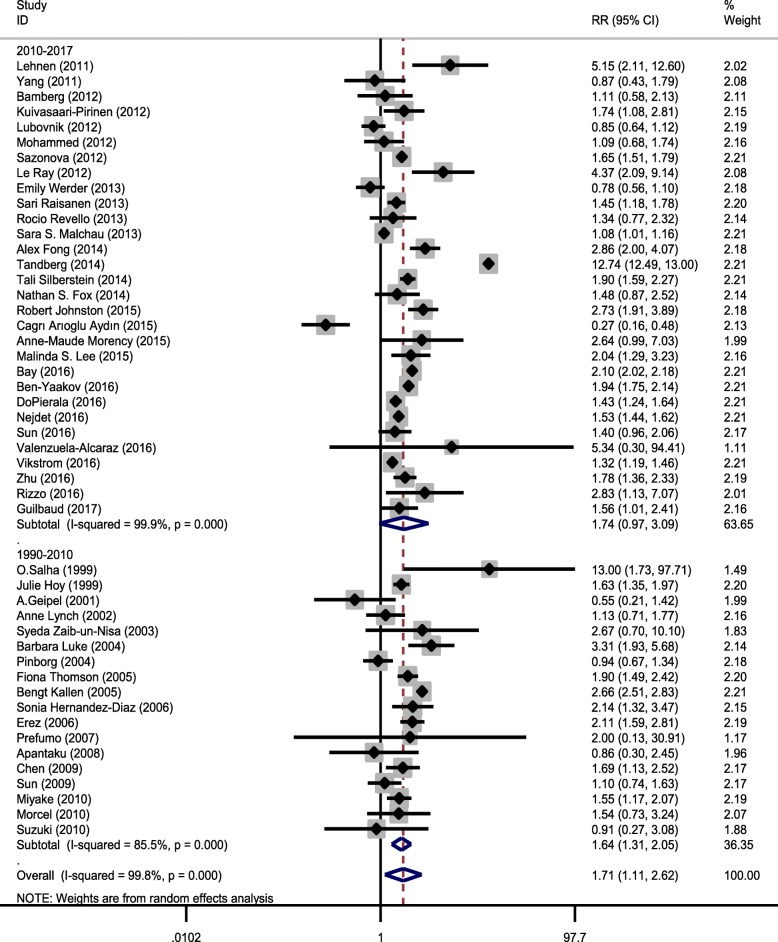
Forest plot showing effect of ART on preeclampsia based on study period

**Fig. 5:**
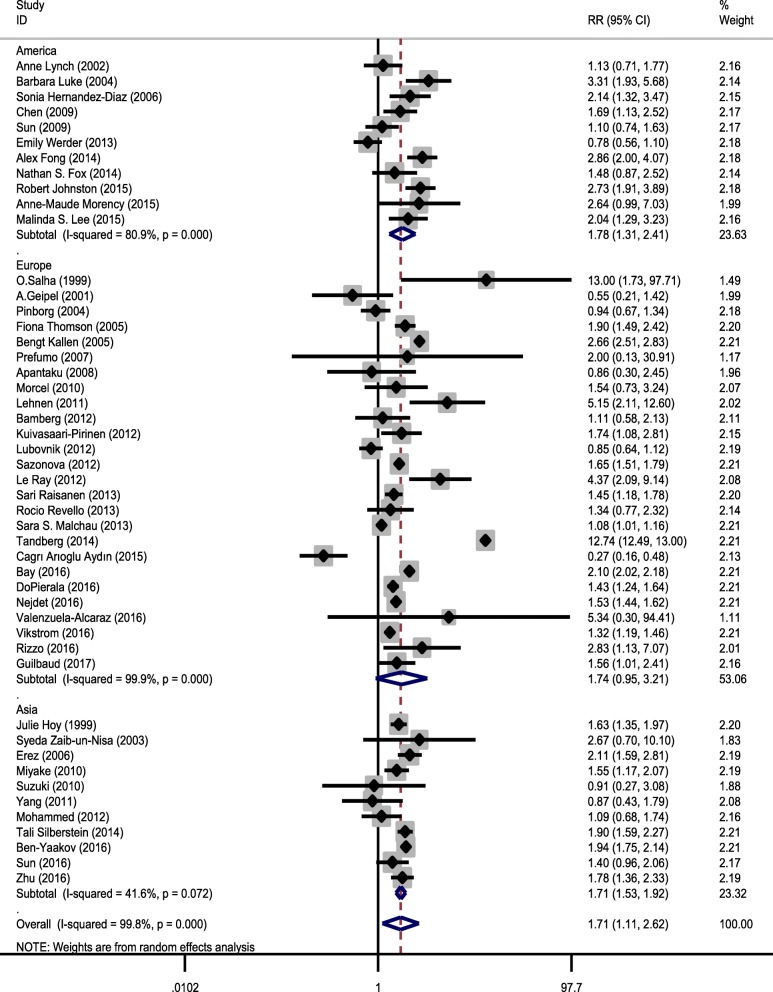
Forest plot showing effect of ART on preeclampsia based on regions

Chi-square analysis showed that there was substantial heterogeneity between primary studies (heterogeneity χ^2^ = 26,313.92, *p* < 0.001, I^2^ = 99.8%, and τ^2^ = 2.17). Therefore, we concluded that the random-effect model was used to pool the studies. To discover the source of heterogeneity, subgroup analysis was carried out on the basis of study design (case control and cohort), study region (United States, Asia, and Europe), and study period (1999–2010 and 2010–2017) (Figs. 3, 4 and 5, and Table 3). After subgroup analysis, heterogeneity across studies did not decrease effectively; therefore, all estimations of RR were made by the random-effect model.

### Risk of publication bias

**Fig. 6:**
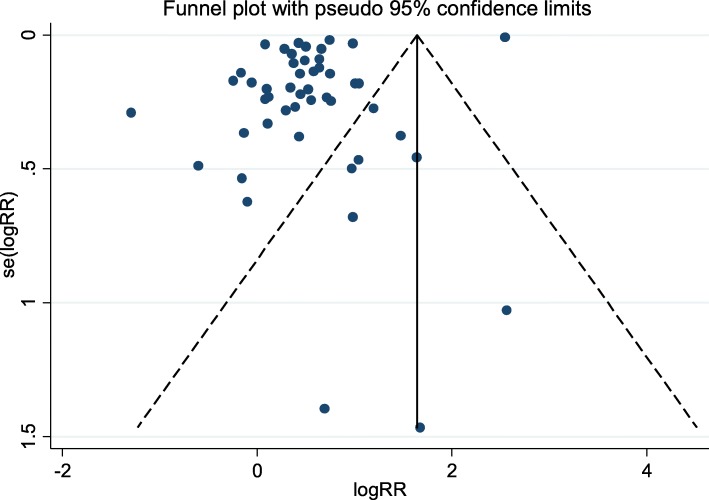
Funnel plots of studies examining the association between ART and preeclampsia

Both graphical and statistical assessments were performed to check for the presence of publication bias. On the basis of the asymmetrical funnel plot (Fig. 6) and Begg’s test (*p* = 0.001), there was evidence of publication bias in this study. Accordingly, we excluded non-English papers from the meta-analysis and this can lead to bias.

## Discussion

This study aimed to evaluate whether several studies agree with the effect of ART on the presence of PE. In this meta-analysis, 6,714,495 cases were recruited (156,246 ART cases and 6,558,249 non-ART cases). To detect the risk of PE regarding the use of ART, the heterogeneity among the studies was assessed, and the appropriate statistical tool was applied. To increase the validity of the results, the risk of publication bias was checked. Analysis of the important subgroups, such as publication date, type of study, and region, was performed.

Similar to the results achieved from our study, most of the studies have introduced the use of ART as a significant risk factor for placental abruption, low and very low birth weight in infants, placenta previa, gestational hypertension, risk of cesarean section, and PE [21, 22]. However, not all the investigators agree with the adverse effect of ART on pregnancy outcomes [23, 24]. Most of previous studies have proven the important impact of using ART on PE [25–28]. The positive association between ART and PE is well demonstrated by the included studies. Regarding the magnitude of the effect size, the pooled results from case-control studies were in compliance with those of cohort studies. However, in contrast to the cohort studies, the pooled RR from the case-control studies was not statistically significant. Moreover, the impact of ART on PE did not differ in two distinct periods of time (2010 as the cut-off point). Although consistent results were observed among different regions, the pooled RR from the European studies was not significant. Moreover, the effect size of the Asian and United States studies was higher than that of Europe.

We found that the use of ART was a significant risk factor for PE. The application of ART has increased across many countries around the world as a way to cope with infertility problems. The prevalence of using ART differs among countries. Annually, more than 1.5% of all births in the United States are the result of ART. The prevalence of PE is almost 10% in Africa and 15% in China [29–32]. In addition, the prevalence of PE has an increasing slope. Numerous factors, including the use of ART, hypertension, diabetes, obesity, and early diagnosis problems, are responsible for the ascending trend of PE prevalence [30, 33]. The adverse outcomes after ART cause damage to body organs, such as the kidney and liver, through PE as well as maternal mortality, perinatal deaths, preterm birth, intrauterine growth restriction, bleeding problems, and fetal growth retardation [34, 35]. In addition to ART, other factors such as anti-phospholipid syndrome, previous PE, family history of PE, insulin-dependent diabetes, obesity, multiple pregnancies, and nulliparity can affect PE [36]. The mechanism in which ART affects PE is not well known. However, it has been argued that abnormal placentation can influence PE. In some ART procedures, the blood flow is compromised and is diminished, which is then followed by obstetric complications. Moreover, placental insufficiency is caused by the transfer of the conceptus into the uterine cavity and the impact of the altered hormonal environment in the endometrium where the development of the maternal–fetal interface can be influenced [37, 38]. It has been argued that ART may have epigenetic effects. The pregnancies from ART are associated with PE through oxidative stress. In addition, ART has several types of reproductive dysfunction with the same strength as miscarriages. Recurrent spontaneous miscarriages, along with infertility treatments, increase the risk of PE in comparison to those without treatment [39]. Nonetheless, the excess RR in the association between ART and PE can be caused by multiple factors, such as previous fertility complications, lifestyle, smoking habits, long inter-birth intervals, multiple pregnancy, and advanced maternal age [39]. However, there are many other causal factors associated with infertility itself in which the relationship between PE and ART can be argued.

Thomopoulos et al. assessed the risk of hypertensive disorders in pregnancy following ART using an overview of the studies conducted from 1978 to 2016 [40]. Their study included papers from PubMed and the Cochrane Collaboration Library databases with a total of 32 papers with PE as an outcome. The present meta-analysis has added primary studies from other databases such as Embase, Scopus, and ISI Web of Knowledge with a total number of 48 papers up to June 2017.

The controversy of using statistical tools to determine the magnitude of heterogeneity in meta-analysis has several potential causes, including sample size and number of the included studies, the period of time, the geographical patterns, the level of development, and the types of studies, etc. In this regard, a non-significant result from a chi-square test must not be taken as evidence of a lack of heterogeneity. Furthermore, the chi-square test is very powerful when many studies are included in a meta-analysis. The other statistical tool to detect heterogeneity, the I^2^ value, depends on the magnitude of the rates [41]. In our meta-analysis, the result of the chi-square test was confirmed by the I^2^ test. Except for a region of Asia, significant heterogeneities were observed among the pooled and subgroup RRs. The source of heterogeneities may be due to the diversity in the ethnic and cultural conditions and uneven development regions.

However, this study has some limitations. Almost every meta-analysis study deals with uncontrolled confounders. Researchers are not able to control the analysis for the confounders unless the proper information is presented by the original articles. To overcome this problem, “individual patient or participant data (IPD)” is suggested in which requires the detailed information and data-sets from every single original article and it is not applicable in most of the cases regarding that the authors (original articles) might not be interested to present their data and other potential reasons.

This systematic review has several limitations. First, the most important limitation for this study as for other systematic review is the lack of data for subgroup analysis based on type of pregnancy (singleton versus twin pregnancy) or for data analysis controlling for known confounders. Second, our study included only English full-text papers. However, globally published papers might present higher quality research compared with those of local origin.

## Conclusion

The present systematic review and meta-analysis revealed that the use of ART increases the risk of PE considerably. More attention must be paid to Asia and the United States, where the association is stronger and significant.

## Abbreviations

ART: Assisted Reproductive Technology
CI: Confidence Interval
PE: Preeclampsia
RR: Relative Risk
